# Assessment of automatic associations with bodily sensations and agoraphobic situations in panic disorder

**DOI:** 10.1016/j.jbtep.2016.04.001

**Published:** 2016-09

**Authors:** Marcella L. Woud, Eni S. Becker, Mike Rinck, Catherine J. Harmer, Andrea Reinecke

**Affiliations:** a Mental Health Research and Treatment Center, Department of Psychology, Ruhr-Universität Bochum, Massenbergstrasse 9-13, 44787 Bochum, Germany; b Behavioural Science Institute, Radboud University Nijmegen, Montessorilaan 3, 6526 HR Nijmegen, The Netherlands; c Warneford Hospital, Department of Psychiatry, University of Oxford, Warneford Lane, Oxford OX3 7JX, UK

**Keywords:** Panic disorder, Automatic associations, Extrinsic affective simon task (EAST), Anxiety, Information processing bias

## Abstract

**Background and objectives:**

One of the central assumptions of cognitive models of Panic Disorder (PD) is that automatic panic-related associations are a core feature of PD. However, empirical findings are mixed and inconsistent, rendering it difficult to evaluate the role of panic-related associations adequately, particularly in relation to the relevant theories. The present study aimed to further advance our understanding of automatic associations in PD, and therefore applied a paradigm novel in this context, namely an Extrinsic Affective Simon Task (EAST).

**Methods:**

Participants involved treatment seeking, unmedicated panic patients (n = 45) and healthy controls (n = 38). The EAST was applied prior to treatment. It included the following stimuli as targets: panic-related bodily sensations and agoraphobia-related situations, and as attributes: pleasant versus unpleasant, fear-related words.

**Results:**

Contrary to our expectations, panic patients did not show stronger negative than positive automatic associations for either panic-related symptoms or agoraphobia-related situations, compared to healthy controls. Moreover, EAST effects did not correlate with panic-related self-report measures.

**Limitations:**

Although the present study involved patients who were actively seeking treatment, panic-related associations might not have been activated sufficiently. Hence, a brief activation procedure (e.g., hyperventilation) might have been needed to optimize the assessment condition.

**Conclusions:**

The present findings do not support contemporary theories of panic-related associations. Therefore, follow-up work is needed to disentangle their functional and operational properties more thoroughly.

## Highlights

• Automatic panic-related associations are a core feature of Panic Disorder.
• We assessed them by means of an Extrinsic Affective Simon Task (EAST).
• We tested panic patients (PP) and healthy controls (HC).
• PP did not show stronger negative automatic panic-related associations than HC.

## 1 Introduction

A central assumption of cognitive models of Panic Disorder (PD) (e.g., Beck et al., 1985, Clark, 1986) is that panic-related associations lie at the heart of PD: They are activated at a very early stage of information processing and occur automatically, i.e., they are activated quickly, unintentionally, and without the individual's control. To illustrate, a PD patient who notices an increase in heartbeat automatically associates this benign bodily sensation with something alarming, resulting in a catastrophic misinterpretation of that sensation (e.g., a heart attack). This is followed by an amplification of bodily sensations, which in turn triggers anxiety and very likely results in a full-blown panic attack. Theoretically, automatic associations are the crucial element here because they explain a patient's inability to deactivate this vicious circle. Furthermore, automatic associations could account for some patient's therapy resistance or relapse. Most interventions target explicit cognitions and this might not necessarily impact automatic associations.

Priming tasks are frequently used reaction time (RT) paradigms to study automatic panic-related associations. Such tasks involve the (brief) presentation of a prime (e.g., a word), followed by a target requiring a response (e.g., categorization). The RT needed to respond to the target serves as an index of the ‘associative match’, i.e., the prime can either facilitate or aggravate reactions and thereby decrease or increase RTs. To illustrate, the priming study by Schniering and Rapee (1997) included associatively related and associatively unrelated prime–target pairs, which participants had to categorize as ‘words’ or ‘non-words’. Most relevant were trials where primes referred to bodily sensations and targets to catastrophic outcomes (e.g., breathlessness-suffocate; dizzy-faint). Contrary to predictions of PD models, there was no difference in RTs between panic patients and controls on panic trials (see also Schneider and Schulte, 2008, McNally et al., 1997). To the best of our knowledge, the study by Hermans et al. (2010) is the first to show the expected pattern, i.e., a faster RTs for panic trials in panic patients than in controls.

Another paradigm is the Implicit Association Test (IAT; Greenwald, McGhee, & Schwartz, 1998). Here, participants sort stimuli (e.g., words) into four categories by means of two response keys: two categories represent a target concept, (e.g., me vs. not me) and two categories represent two poles of an attribute dimension (e.g., panicked vs. calm). Each target category is paired with both attributes. As such, faster RTs during a particular target–attribute combination suggest a strong association between the two stimuli. To illustrate, the study by Teachman, Smith-Janik, and Saporito (2007) found that panic patients, compared to healthy controls, had stronger associations between concepts related to ‘me and panicked’ than between ‘not me and panicked’. However, a second IAT using the concepts ‘bodily changes versus body parts’ and ‘alarming versus meaningless’ did not reveal any group differences.

To conclude, findings concerning automatic, panic-related associations are mixed. Various reasons could account for this. For example, the tested samples differed in panic-related severity, and panic-related associations might have been more accessible in those patients with more severe symptoms. Furthermore, the stimuli used differed in their ecological validity and results partly depended on analyses using idiographically selected stimuli (Schneider & Schulte, 2007). This inconsistency makes it difficult to evaluate the role of panic-related associations adequately. Hence, the present study aimed to extend previous findings by assessing automatic panic-related associations using a novel paradigm in the context of PD, namely the Extrinsic Affective Simon Task (EAST; De Houwer, 2003). During the EAST, attribute words are categorized by means of two response keys, assuming that the keys become extrinsically associated with the attributes' valence. In contrast, target words have to be categorized by means of a task irrelevant feature, (e.g., color), using the same two response keys as during attribute categorization. The associative strength is defined via the RT difference between giving a pleasant versus unpleasant response to a target (for other EAST studies, see e.g., Ellwart et al., 2005, De Raedt et al., 2006, Roefs et al., 2005). In the present study, the EAST was applied in a sample of clinically diagnosed panic patients and healthy controls (attributes: pleasant and unpleasant words, targets: panic-related bodily sensations and agoraphobia-related situations).

Compared to previous studies, our study has a number of advantages. First, as PD does not have an inherently meaningful contrast category (which previous IAT studies needed), our critical test concerns the automatically associated valence comparison. Second, the EAST employs a task-irrelevant instruction. Hence, participants respond to stimulus features that are independent of the stimulus dimension the task aims to assess (compared to, for example, the IAT), disguising the research question and making response strategies less likely (Rinck & Becker, 2007). Third, we recruited a non-biased control group, i.e., a group that was not exposed to panic-related information, and therefore could not have obtained a panic-related bias which could impact RT effects. To illustrate, Hermans et al. (2010) control group partly included professionals working within the health service. Hence, these participants had a basic knowledge of panic-related phenomena, which could have affected the priming task's results. Given these advantages, our study offers new and advanced insights to the role of automatic associations in PD. Moreover, if successful, variations of the EAST could provide a useful starting point to systematically examine reasons that could account for the previous inconsistent findings (e.g., by comparing different sets of stimuli).

We expected panic patients, compared to controls, to show stronger negative than positive automatic associations for both panic-related symptoms and agoraphobia-related situations. Moreover, we expected the EAST effects to be correlated with panic-relevant self-report measures.

## 2 Methods

### 2.1 Participants

85 participants were tested. Two participants were excluded due to missing data (final sample: *N* = 83). There were *n* = 45 panic patients (7 male, 34 female, *M*_*age*_ = 32, *SD* = 11; PD without agoraphobia *n* = 15, PD with agoraphobia *n* = 30), recruited from an outpatient waiting list and diagnosed using the Structured Clinical Interview for DSM-IV Axis I Disorders (SCID-CV; First, Spitzer, Gibbon, & Williams, 1996). Currently being on CNS-active medication such as antidepressants and comorbidity were exclusion criteria (Reinecke & Harmer, 2016). Patients were tested before their first treatment session. The control group included *n* = 38 participants (4 male, 32 female, *M*_*age*_ = 31, *SD* = 11), without current or history of psychopathology, recruited via newspapers and posters. There were no group differences in age, *t*(81) = .47, *p* = .638, or gender, χ^2^(2) = .96, p = .618.

### 2.2 Questionnaire measures

#### 2.2.1 Panic disorder severity scale (PDSS; Houck, Spiegel, Shear, Rucci, & Stat, 2002)

This 7-item self-report scale measures severity of PD, assessing, for example, distress during panic attacks and panic frequency.

#### 2.2.2 Agoraphobic cognitions questionnaire (ACQ; Chambless, Caputo, Bright, & Gallagher, 1984)

The ACQ includes 14 items and measures dysfunctional cognitions in relation to potential catastrophic consequences arising from panic or anxiety using two subscales: loss of control and physical concerns.

#### 2.2.3 Hospital anxiety and depression scale (HADS; Zigmond & Snaith, 1983)

The HADS measures the severity of anxiety-related and depression-related symptomatology. It consists of 14 items, half of them related to anxiety and the other half to depression.

#### 2.2.4 Trait anxiety inventory (STAI-T; Spielberger, Gorsuch, Lushene, Vagg, & Jacobs, 1983)

The STAI-T was used to assess trait anxiety. It comprises 20 anxiety related statements that participants rate for occurrence and frequency.

### 2.3 Extrinsic affective simon task (EAST)

Targets were 10 words describing panic-related bodily sensations and 10 words describing agoraphobia-related situations. Each of the target words had a blue-colored and a green-colored version. Moreover, 10 pleasant and 10 unpleasant black-colored valence words were used (for an overview, see Table 1).

### 2.4 Procedure

The EAST started with two practice blocks. In the *valence practice block*, participants categorized single black printed words as either “pleasant” or “unpleasant” by pressing a left or a right response key. By doing so, the response keys became extrinsically associated with the meaning of the valenced stimuli assigned to them (valence and key position was counterbalanced across participants). Each word was presented three times and in random order (60 trials in total). In the *color practice block*, colored words appeared on the screen, and participants had to categorize them based on their color. The neutral practice words were printed in either blue or green. During the color practice block, participants had to use the same keys as during the valence practice block. All participants were instructed to use the left key to respond to green words, and the right key to respond to blue words. Each of the neutral practice words was presented twice, once in blue and once in green (20 trials in total).

Subsequently, the *experimental blocks* started. There were four blocks consisting of 60 trials each (240 trials in total, trials were pseudo-randomized within each block). In one third of the trials, a black-printed *valence word* (valence trials) was presented. These words had to be categorized as either pleasant or unpleasant, in order to maintain the association of the response keys. In the remaining two thirds, a blue or green colored *target word* was presented (target trials), requiring a categorization with respect to the words' color. There were two types of targets, i.e., bodily sensation targets and agoraphobic situation targets. Each target was presented 8 times, during which the target words appeared 4 times in green and 4 times in blue. Thus, participants had to respond to panic sensation target words with the “unpleasant” key versus with the “pleasant” key in half of the trials. Equally, they also responded to agoraphobic situation target words with the “unpleasant” key versus with the “pleasant” key in the other half of the trials. Two types of experimental conditions were therefore created: A PD-consistent condition, whereby panic-related bodily sensations and agoraphobia-related situations had to be categorized with the “unpleasant” key, and a PD-inconsistent condition, whereby panic-related bodily sensations and agoraphobia-related situations had to be categorized with the “pleasant” key. Following the EAST, participants completed the self-report questionnaires. All participants received a small monetary reimbursement for their time and effort.

## 3 Results

### 3.1 Clinical self-report measures

Independent t-tests showed that there were significant group differences on all measures (PDSS, ACQ, HADS, and STAI-T), *t*'s(81) > 10, *p*'s < .001, with panic patients scoring higher than controls (for means and standard deviations, see Table 2).

### 3.2 Extrinsic affective simon task (EAST)

**Fig. 1:**
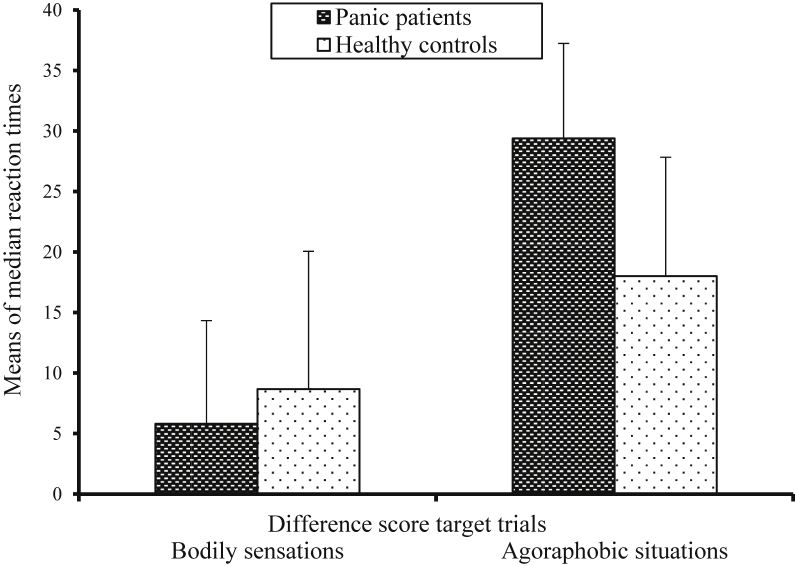
Mean RTs for panic patients and healthy controls for the 4 different scores. Difference scores were calculated for each target type (i.e., one for bodily sensations and one for agoraphobic situations) by subtracting the median RT of the “pleasant key” from the median RT of the “unpleasant key”. Error bars represent standard errors.

Overall error rates were low (*M* = 4.74, *SD* = 5.04) and there were no group differences in error rates (*t* = .28, *p* = .78; panic patients: *M* = 4.6, *SD* = 5.38, controls: *M* = 4.92, *SD* = 4.66). Prior to the analysis, EAST effects (i.e., difference scores) were generated from correct target trials (for a similar procedure, see e.g., Reinecke, Rinck, Becker, & Hoyer, 2013). Two differences scores were calculated, i.e., one for each target type, by subtracting the median RT of the “pleasant key” from the median RT of the “unpleasant key”. As such, a negative difference score indicates a negative association and a positive difference score a positive association with the respective target type (for means and SDs of raw response data, see Table 2). Following this, the analysis is based on a two-factorial design with within-subjects factor Target Type (difference score bodily sensations, difference score agoraphobic situations) and between-subjects factor Group (panic patients, healthy controls). Results of a repeated-measures ANOVA showed that there was a significant main effect of Target Type, *F*(1,81) = 6.05, *p* < .02, eta^2^ = .07, with means indicating that overall, participants' association towards agoraphobia-related situations was more positive than towards panic-related bodily situations (agoraphobic situations: *M* = 24.18, *SD* = 56.41, bodily sensations: *M* = 7.13, *SD* = 63.1). The main effect of Group was not significant, *F*(1,81) = .14, *p* = .711,, eta^2^ = .002. Of greatest interest was the Target Type × Group interaction. However, this interaction was not significant, *F*(1,81) = 1.13 *p* = .29, eta^2^ = .02, showing that panic patients, compared to healthy controls, did not show stronger negative than positive automatic associations for panic-related symptoms and agoraphobia-related situations (panic patients: bodily sensations: *M* = 5.81, *SD* = 57.2, agoraphobic situations: *M* = 29.39, *SD* = 52.75; controls: bodily sensations: *M* = 8.68, *SD* = 70.21, agoraphobic situations: *M* = 18.01, *SD* = 60.59, see Fig. 1).^1^

### 3.3 Correlations

Correlational analyses were conducted among the entire sample and separately for both groups. Of main interest were the correlations between the EAST effects (i.e., the two difference scores for the two target types) and the clinical self-report measures among panic patients. However, among the group of panic patients, analyses revealed a significant correlation only between agoraphobic situations and the STAI-T, *r* = −.38, *p* < .01, indicating that less positive associations towards agoraphobic situations were related to higher trait anxiety (for a complete overview, see Table 3).

## 4 Discussion

The present study examined the role of automatic associations towards panic-related bodily sensations and agoraphobia-related situations by means of an EAST. However, results did not confirm our expectations. First, there was no group difference in EAST difference scores. That is, panic patients did not show stronger negative, automatic associations towards panic-related bodily sensations and agoraphobia-related situations than healthy controls. This, in combination with the general mixed findings in this context, makes it difficult to evaluate the role of automatic, panic-related associations in relation to the underlying theory. Hence, on the one hand, future research is needed to continue testing predictions of cognitive models in PD. On the other hand, present theoretical approaches may need to be refined. Correlational analyses showed that EAST effects were not associated with panic-relevant self-report measures. A limitation of the present study is that it did not include a procedure to activate panic-related associations (e.g., hyperventilation), which might have been needed for a full activation of the panic-related memory schemata.

Following our and previous studies, it seems safe to conclude that the examination of automatic, panic-related associations depends on subtle experimental conditions. What should future research target? First, it should aim to integrate physiological measures. As Hermans et al. (2010) proposed, it is possible that panic-related cues elicit negative associations in both panic patients and non-patients. However, physiological responses towards these cues could differentiate between the groups. Second, there is little work addressing individual differences in automatic, panic-related associations. The results of Teachman, Marker, and Smith-Janik (2008) showed that not all panic patients experienced a reduction in panic-related associations from pre to post treatment. This raises the important question of whether such associations are indeed an etiological factor. Third, PD is characterized by a number of dysfunctional cognitive processes, e.g., in interpretation (Woud, Zhang, Becker, McNally, & Margraf, 2014). However, there is little research on the relation and interplay of these processes (for an exception, see e.g., Teachman et al., 2007).

Taken together, our data did not support a strong role of automatic, panic-related associations in PD. Nevertheless, these results provide key hypotheses and follow-up research is clearly warranted.

## Role of funding organizations

This research was supported by a Medical Research Council (MRC) grant awarded to Catherine Harmer (G0501223) and a fellowship of the German Research Foundation (DFG) (RE 2698/2-1) awarded to Andrea Reinecke. The MRC/DFG had no role in study design; in the data collection, analysis, and interpretation of data; in the writing of the paper; and in the decision to submit the article for publication.

## Conflict of interest

None of the authors have any conflict of interest with respect to the contents of this paper.

## Figures and Tables

**Table 1:** Overview stimuli EAST.

Bodily sensations	Agoraphobic situation	Unpleasant	Pleasant
Numbed	Museum	Dangerous	Lovely
Dizzy	Restaurant	Fear	Pleasure
Tremble	Railway	Panic	Pleasant
Nervousness	Bus	Threat	Delight
Breathlessness	Theatre	Anxiety	Beautiful
Sweat	Lift	Shock	Happy
Sickness	Aeroplane	Horrify	Glad
Palpitation	Shop	Anxious	Happiness
Confusion	Tunnel	Frightened	Fun
Heartbeat	Boat	Harm	Joyous

**Table 2:** Means and standard deviations of the clinical self-report measures and EAST.

	Panic patients (*n* = 45)	Healthy controls (*n* = 38)
Dependent measure	*M (SD)*	*M (SD)*
PDSS	11.07 (5.75)	.08 (.36)
ACQ	2.34 (.56)	1.30 (.23)
HADS-A	13.42 (3.77)	3.95 (3.38)
HADS-D	7.87 (4.00)	.92 (1.22)
STAI-T	56.09 (9.76)	33.47 (8.29)
Bodily sensation – pleasant	653.41 (124.47)	593.22 (93.54)
Bodily sensation – unpleasant	659.22 (110.05)	601.91 (101.99)
Agoraphobic situation – pleasant	621.03 (102.82)	554.76 (64.09)
Agoraphobic situation – unpleasant	650.42 (113.27)	572.78 (93.87)

*Note*. PDSS = panic disorder severity scale; ACQ = agoraphobic cognitions questionnaire; HADS-A = hospital anxiety and depression scale - anxiety; HADS-A = hospital anxiety and depression scale - depression; STAI-T = trait anxiety inventory; Bodily sensation – pleasant: RTs bodily sensation target trials combined with pleasant key; Bodily sensation – unpleasant: RTs bodily sensation target trials combined with unpleasant key; Agoraphobic situation – pleasant: RTs agoraphobic situation target trials combined with pleasant key; Agoraphobic situation – unpleasant: RTs agoraphobic situation target trials combined with unpleasant key.

**Table 3:** Correlations among panic patients, healthy controls and total sample for EAST effects and clinical self-report measures.

Group	Measure	1.	2.	3.	4.	5.	6.
Panic patients	1. EAST bodily sensations	–					
	2. EAST agoraphobic situation	.44**	–				
	3. PDSS	.06	-.11	–			
	4. ACQ	-.14	-.12	.39**	–		
	5. HADS-A	.01	-.22	.56**	.43**	–	
	6. HADS-D	.04	-.13	.48**	.42**	.59**	–
	7. STAI-T	-.07	-.38**	.32*	.45**	.52**	.44**
Healthy controls	1. EAST bodily sensations	–					
	2. EAST agoraphobic situation	.54**	–				
	3. PDSS	-.03	-.05	–			
	4. ACQ	.1	-.23	.27	–		
	5. HADS-A	.15	-.09	.41*	.62**	–	
	6. HADS-D	.06	.08	.2	.28#	.57**	–
	7. STAI-T	.3#	.21	.04	.53**	.63**	.49**
Total sample	1. EAST bodily sensations	–					
	2. EAST agoraphobic situation	.49**	–				
	3. PDSS	.0	.03	–			
	4. ACQ	-.06	-.01	.75**	–		
	5. HADS-A	.03	-.01	.8**	.79**	–	
	6. HADS-D	.0	.03	.78**	.75**	.81**	–
	7. STAI-T	.05	.0	.72**	.78**	.84**	.76**

Note: Panic patients: N = 45; Healthy controls: N = 38; Total sample: N = 83; EAST = extrinsic affective simon task; PDSS = panic disorder severity scale; ACQ = agoraphobic cognitions questionnaire; HADS-A = hospital anxiety and depression scale - anxiety; HADS-A = hospital anxiety and depression scale - depression; STAI-T = trait anxiety inventory;*p < .05, **p < .01, #*p* < .1.

## Footnotes

1 When using d-scores results did not change.
